# Perceived utility of an integrated psychological intervention for gynaecological cancer patients admitted for surgery: preliminary data

**DOI:** 10.3332/ecancer.2017.722

**Published:** 2017-02-23

**Authors:** Paola Arnaboldi, Serena Oliveri, Valeria Vadilonga, Luigi Santoro, Angelo Maggioni, Gabriella Pravettoni

**Affiliations:** 1 Applied Research Division for Cognitive and Psychological Science, European Institute of Oncology (IEO), Via Ripamonti, Milan, Italy; 2 Division of Epidemiology and Biostatistics, European Institute of Oncology (IEO), Via Ripamonti, Milan, Italy; 3 Department of Oncology and Hemato-Oncology (DIPO), University of Milan, Via Festa del Perdono, Milan, Italy; 4 Gynecology Oncology Division, European Institute of Oncology (IEO), Via Ripamonti, Milan, Italy

**Keywords:** gynaecological cancer surgery setting, psychological intervention, interprofessional practice, cancer patients’ distress, patient satisfaction

## Abstract

**Objective:**

To investigate patients’ satisfaction and perceived utility for psychological consultations delivered by clinical psychologists in a sample of gynaecological cancer patients hospitalised for surgery.

**Methods:**

A total of 51 gynaecological cancer patients who scored higher than four on the distress thermometer (DT) were proposed and received a psychological consultation during hospitalisation for surgery. After six months from discharge, patients were asked, during a telephone interview, to rate their level of distress post-treatment, their perceived satisfaction, and usefulness of the psychological intervention received.

**Results:**

At the time of the telephone interview, the distress levels stated by patients tended to be lower than those at hospital admission, and around 61% of the patients expressed maximum satisfaction with psychological intervention. Among these, 60.8% rated the psychological consultation useful for dealing with the hospitalisation itself, 45.1% useful for dealing with personal issues and 58.8% for dealing with issues related to returning home. People who were at their first diagnosis and those who had no other reason to be distressed beyond their cancer found psychological support significantly more useful for facing up to personal issues.

**Conclusions:**

Patients are highly satisfied with integrative psychological interventions delivered by clinical psychologists in a medical setting such as that of gynaecological cancer surgery and from the six-month follow-up, it emerged that such interventions help in promoting patients’ adjustment to the phase of hospitalisation and post-hospital discharge.

## Introduction

National and international guidelines have been developed to detect, identify, and manage the psychosocial distress and related needs of people with cancer by means of validated instruments that healthcare delivery organisations should integrate into their routine care [1–3].

In 2011, Bultz *et al.* [4] posited that psychological distress must be considered as the sixth vital sign together with blood pressure, body temperature, pulse (heart rate), breathing rate (respiratory rate), and pain level.

Evidence from randomised trials shows that psychologically effective interventions may lead to a survival advantage and to an improvement of quality of life in patients with cancer [5, 6]. Unfortunately, no clear statements can be made regarding the types of therapists who can bring about these gains, since their training has been seen to range from undergraduate degree to doctoral level with several disciplines represented (e.g., psychology, psychiatry, nursing, and social work) [7]. A consensus has not yet been reached upon how to integrate psychosocial care into the multidisciplinary model of assistance nor upon which professionals should deliver such interventions [8–12].

In the literature, the effectiveness of psychological intervention is observed in terms of changes in the prevalence of specific distressing symptomatology such as emotional distress, anxiety, and depression [13] or in terms of changes in adherence to the process of care and survival [14–22]. However, patient satisfaction is not mentioned as one of the primary parameters of effectiveness.

On the other hand, there are different levels and significance of distress in health care and not all can be addressed in the same way by the same healthcare professional: the issues to address can range from normal numbness and sadness, through increased risk of vulnerability to distress, poor sleep, poor appetite preoccupation with thoughts of illness and death, concerns about social role, adjustment disorders, and significant symptomatology of depression and anxiety. The National Institute for Clinical Excellence [3] recommended a four-level model for psychological assessment and support: liaison psychiatry and clinical psychology (level 4), registered counsellors (level 3), clinical nurse specialists in cancer care, chaplains, general practitioners, and other palliative care specialists (level 2). Level 1 represents the psychological support, which all health and social care staff could or should be able to provide.

Among gynaecological cancer patients, the presence of distress is seen as high with clinically significant depression and anxiety prevalence being at around 30% [23]. The prevalence of pathological anxiety among ovarian cancer patients is about 74% [24, 25] and worry constitutes an unmet need in 70.7% of cervical cancer patients and in 34.7% of endometrial cancer patients before surgery [26, 27]. In a study by Mehnert *et al.* [28], the prevalence of any mental disorders among gynaecological patients ranges from 28.94% to 43.21%. A number of risk factors, such as demographic data, medical conditions and personal predispositions [29], along with sexual, body image disorders and relationship problems [30, 31], have been demonstrated to predict poorer adjustment along cancer trajectory in this population of cancer patients.

The aim of the present study was to investigate the reported levels of satisfaction with the psychological intervention delivered by clinical psychologists to a sample of gynaecological cancer patients before surgery and to quantify the perceived utility of the intervention in promoting adjustment to the illness process as reported by patients at six-month follow-up.

## Materials and methods

### IEO policy and screening of psychological distress

According to national and international guidelines [1–3], each patient accessing European Institute of Oncology (IEO) clinical pathways, either in the medical or in the surgical area, must be screened for the presence of psychosocial distress. For this reason, the IEO utilises the Italian version of the distress thermometer (DT) [32]. Through this tool, the patient is asked to describe the amount of emotional distress experienced in the last week, indicating a number ranging from 0 (no emotional distress – no stress) to 10 (maximum emotional distress – maximum stress) in a drawn thermometer. The instrument is usually administered to patients by nurses or, when time is not available, it is self-administered by patients, who must fill it in by themselves.

Patients with a score of distress higher than 4 [32] are referred to the IEO psychology division to receive the intervention of a specialised psychologist.

### Distress screening policy and gynaecological cancer patients

In the gynaecological ward, cancer patients enter the waiting list after being booked to undergo a surgical procedure. During the one-day pre-hospital admission (about 4 weeks before surgery), the case manager nurse administers the distress thermometer to the patient and, when high distress is registered, she requests the psychology division to intervene.

We need to specify that about 70% of IEO patients live far from the hospital [33], and the first psychological face-to-face consultation is usually performed at hospital admission because of the physical distance and organisational aspects that make it unfeasible to perform psychological consultation at pre-hospital admission.

Furthermore, patients might refuse psychological intervention at pre-hospital admission, but usually, they ask for it at admission for surgery. The number of psychological intervention sessions depends on the type of surgical procedure, on hospitalisation length and on personal issues, and it ranges from a minimum of one focused session to a maximum of eight sessions, usually devoted to pelvic exenteration patients [34].

### The model for psychological consultation

Consultations are delivered by licensed clinical psychologists with 10 years’ experience in the psycho-oncology field. They had a four-year specialisation degree in integrated psychotherapy. This means that they had a technical and theoretical background either in cognitive–behavioural, in psychodynamic and existential psychological approaches. Matching psychological interventions to the specific psychological functioning of the patient in every therapeutic phase is the basis of integrated psychological approaches. The accumulating research demonstrates that it is indeed frequently effective to tailor psychological intervention to the entire person and to his or her own issues beyond his or her disorder [35].

**Figure 1.**
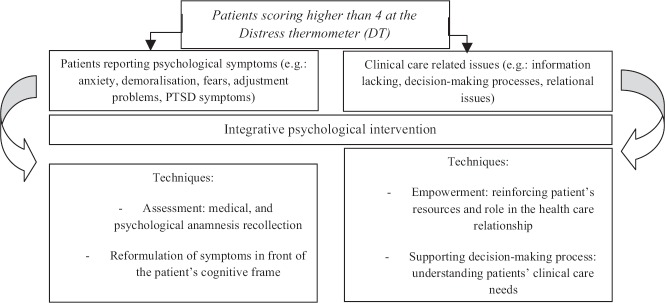
Development of the integrative psychological intervention.

On the basis of the tailoring principle, in our sample, the psychological intervention focused on two different issues with the application of different techniques as described in Figure 1: in the first case, the intervention consisted in a psychological assessment and medical history recollection followed by a re-formulation of the patient’s psychosocial and clinical situation (assessment and clinical intervention). This procedure is usual when patients have specific psychological symptoms, such as anxiety or mood disturbances, related to a complex psychological and psychiatric anamnesis as well.

In the second case, the psychologist focused the intervention on patient’s empowerment and mobilisation of coping resources. This intervention takes place for patients experiencing some kind of problems with the relational aspects of being a cancer patient: relational problems with physicians and/or nurses, decision-making issues, lack of comprehension and information about their clinical situation, and difficulties in managing contacts with the multidisciplinary team members (empowerment intervention focused on clinical care processes).

Patients were initially offered a single-session consultation with the possibility to extend it to a maximum of eight sessions on the basis of patient’s psychological distress and hospitalisation length.

Each consultation is preceded and followed by a discussion with the patient’s primary nurse, to guarantee high focus on clinical and care issues and the integration of psychological intervention into the multidisciplinary team action. The intervention demonstrated to be feasible in gynaecological cancer setting because of its complexity in particular before surgery when patients with psychological suffering confront themselves either with psychological symptoms and/or with clinical-care focused difficulties. The primary endpoint of the intervention was to address patients’ psychological distress by means of a psychological work tailored on each single patient. The secondary endpoint was to develop an intervention perceived as useful by patients, as regards to hospitalisation and recovery issues.

The average consultation time is 45 minutes and the setting is a reserved office located in the gynaecological oncology surgical ward.

### Study procedure and measures

From January 2015 to June 2015, each patient admitted to the IEO gynaecological cancer ward to undergo a surgical procedure was screened in order to detect clinically significant psychological distress by means of the distress thermometer (DT). All the gynaecological patients who scored higher than 4 to the DT received psychological intervention and were enrolled for the following study, none excluded. After 6 months from the hospital discharge, a designated psychologist contacted patients by telephone asking for their consent to undergo a telephone interview about their satisfaction and their perceived usefulness with the psychological consultation received during recovery for surgery. Patients were asked to rate their perceived satisfaction and usefulness on the basis of a 10-point visuoanalogue scale, ranging from 0 (‘not satisfied, at all’) to 10 (‘absolutely satisfied’), and then specifying for what aspect they considered the psychological intervention useful or satisfying. Since 72.6% of patients were at their first diagnosis, we considered a 6-month follow-up to be a reasonable period, having completed their adjuvant treatment and presumably having started their adjustment process to the illness trajectory.

Patients were also asked to recall their cancer experience up to that point, the level of psychological distress they had at the time of hospitalisation for surgery and the role psychological consultation had in managing that distress. They were then asked to evaluate the level of distress perceived in the week preceding the phone interview and to evaluate whether or not it was related to cancer and its sequelae.

### Statistical methods

Descriptive statistics were used to describe the sociodemographic data, type of tumour, and clinical characteristics of participants. A dependent *t*-test analysis was performed to evaluate significant differences in patients’ distress pre- and post-surgery. Significant differences in patient distress (DT scores) were calculated using the Kruskal–Wallis test or Wilcoxon’ test considering patients’ characteristics (e.g.. age, first diagnosis vs. recurrence, type of diagnosis etc. tab. 1) as independent variables, either pre-surgery and post-surgery. Non-parametric analysis Pearson’s chi-square test, Kruskal–Wallis test or Wilcoxon’ test were calculated to evaluate differences in satisfaction and utility perception (measured on the 10-point visuoanalogue scale) at 6-month follow-up after psychological intervention, considering patients’ personal characteristics (e.g. age, worries with cancer vs. other that cancer), experience with cancer (recurrence, type of diagnosis) and type of intervention (assessment vs. empowerment) as independent variables.

Bonferroni correction for multiple comparisons was applied.

All the analyses were performed with SPSS statistical software Version 19.0.

## Results

Since the beginning of our study, 185 patients were admitted to the gynaecological ward for surgical procedures. Among them, 51 patients (year of first diagnosis between 2003 and 2015) scored higher than four at the distress thermometer and received a psychological consultation. Patients’ characteristics are described in Table 1.

A percentage of 48.0 % of patients was diagnosed with ovarian cancer, 30% with cervical cancer, 10.0% with vulvar cancer.

On admission for surgery, 72.6% of patients were at their first diagnosis.

A total of 51% of the patients received a single consultation during admission while 49% received two or more consultations (Table 1). In 64% of the cases, the intervention focused on assessment and anamnestic recollection followed by a reformulation of the given psychosocial and clinical situation, while in 36% of the cases, it focused on empowerment and mobilisation of coping resources (Table 1). No differences were found between patients undergoing the two different types of intervention. Eight patients were not able to participate in the 6-month follow-up: four of them died from disease progression and four were telephonically unavailable.

### Distress and patients’ self-reported outcomes

The mean value for distress at hospital admission was 7.69 while at 6-month follow–up, it was 6.95 (values ranging from 0 to10 on DT). Distress tended to diminish (Δ = −0.8 ± 3.20), although this reduction was not statistically significant (*T*(43) = 1.56; *p* = 0.12).

Patients’ mean score of satisfaction with the consultation received during hospitalisation, evaluated at the 6-month follow-up was 9 (values ranging from 0 to 10 on the visuoanalogue scale). Around 61% of the patients scored 10 (maximum satisfaction on the visuoanalogue scale). Among these, 60.8% rated the psychological consultation useful for dealing with the hospitalisation, 45.1% useful to deal with personal issues and 58.8% to deal with returning home.

### Subgroup analysis

Significant differences were found in distress levels pre-surgery between patients at the first diagnosis versus those experiencing a recurrence (*W* = 259.5; *p* = 0.02). In addition, a statistically significant difference in distress levels at hospitalisation (*W* = 521.0; *p* = 0.007) was reported between the patients who did not have any concern other than carcinoma versus those having other reasons to be distressed (Table 2). People who had other concerns besides cancer were significantly higher in distress levels after surgery. No significant differences were found for satisfaction of psychological intervention among the same groups described in Table 2.

Regarding the perceived utility of psychological consultation, 60.8% of the patients considered the consultation useful to face up hospitalisation and 37.2% of them rated it ‘very useful’ (score 9 or 10) on the visuoanalogue scale. About 45.1% of the patients evaluated the psychological consultation useful to face up personal issues, with 27.4% judging it ‘very useful’ (9 or 10 on the visuoanalogue scale), and 58.8% evaluated the consultation useful to face up returning home, and 27.4% considered it very useful .

In Table 3, the subgroup analyses according to the perceived utility of the consultation are reported. Results show that people who were at their first diagnosis found psychological support significantly more useful for elaborating personal issues (*X*^2^(2) = 8.90; *p* = 0.01). The same was for patients who had no reason other than cancer to be distressed: they were psychologically distressed by the elaboration of contents linked with the cancer disease, so they considered psychological intervention significantly more useful to face up to these specific issues (*X*^2^(2) = 7.13; *p* = 0.02). There were no other significant differences between groups of patients in the evaluation of what the psychological intervention had been useful for (addressing the issues hospitalisation, personal content and returning home). Overall, all patients’ groups assigned to the psychological intervention a utility value no lower than 8/10 (labelled as ‘sufficiently useful’).

## Discussion

As declared in the regulation statements and guidelines established by the National Institute for Clinical Excellence [3], taking charge of the psychological suffering in medical settings involves different professional roles. The American Psychological Association (APA) identifies psychologists as the leading professionals developing evidence-based psychological treatments for cancer patients and having specialised training in assessing, monitoring, and treating cancer psychological sequelae [36]. Nevertheless, in multidisciplinary clinical practices, we argue that questioning patients’ perception of psychological consultation delivered by specialists is needed and represents the first step towards understanding how effective each intervention with its distinctive characteristics actually is. This process is fundamental in the light of the interdisciplinary model, which sees the contribution of expertise from different disciplines as the main source in interprofessional practice for cancer care [22]. It is also warranted from a personalised medicine perspective [37] in which psychological status is a fundamental aspect of patient’s well-being and recovery.

One of the most important pieces of evidence of the present study is that patients recognise the effectiveness of psychological intervention in the context of the very time of the surgical procedure for gynaecological cancer. Data show that a targeted consultation, based on the integrative psychological approach devoted to gynaecological cancer patients and conducted by licensed psychologists with a 4-year specialisation degree in integrative psychotherapy, can promote, as directly reported by patients, their resources to deal with the phase of hospitalisation, to face up personal issues in the cancer journey and to face up to returning home after surgery. Indeed patients expressed full satisfaction with the consultation received during hospitalisation (mean score 9) and about half of them rated it ‘very useful’ to face up to hospitalisation, personal issues (mainly highlighted by people who were at their first diagnosis and focused on cancer in particular) and returning home.

Hence, promoting empowerment in these areas during hospitalisation is strictly warranted and healthcare providers should be attentive to psychological factors both during active treatment and in long-term survivorship.

Our data show a trend of decreasing distress in the gynaecological patients between the surgical period and the 6-month follow-up. This could be related both to the psychological intervention received, as claimed by the patients themselves, and to the passage of time, which leads to a physiological adjustment process to the illness. The levels of distress experienced by patients during hospitalisation, however, seem largely related to the time of diagnosis, with patients at the first diagnosis experiencing more distress than those facing a relapse). Distress levels are also related to the personal context, with patients who also have personal issues, other than cancer, experiencing higher distress than patients who are focused on cancer concerns). Thus, the time of first diagnosis and patient’s subjective reaction together with patients’ medical history should be thoroughly investigated and supported before and during hospitalisation by professional experts, since the presence of concomitant personal stressful events have been shown to influence the distress experienced [29, 34].

We would argue that our evidence might contribute to define proper policy aimed at addressing psychological distress in medical settings such as that of cancer surgery.

As part of the future clinical pathway at IEO, we are defining a new intervention policy that includes a mixed intervention comprising both telephone and face-to-face psychological consultation, in order to ensure care of patients’ distress right from pre-hospital admission, thereby overcoming barriers related to patients’ physical distance from the comprehensive cancer centre.

Furthermore, we consider it important to compare psychological interventions delivered by professional psychologists, and, in particular, our integrative model of intervention, to interventions delivered by other healthcare professionals such as nurses or counselors in order to create recommendations for best practice by means of randomised controlled trials.

One of the most important study limits is the lack of a control group. Other potential limitations of our study might be: the small sample size that reduces the power of our analyses; the relationship between disease stage (local v advanced disease) was not examined and it is possible this would have impacted distress levels and/or response to the intervention; sources of distress other than cancer were not collected at the first assessment which may also have impacted distress levels and/or response to the intervention. These aspects require further research.

## Conclusion

The paradigm of the patient-centred approach in medicine highlights the importance of integrating the management of psycho-social factors from the very beginning of the care delivery process. By means of the present study, we aimed to provide preliminary evidence of the perception patients have about the psychological interventions delivered by professional psychologists in the medical setting of gynaecological cancer surgery. Using these preliminary results about satisfaction and utility as a starting point, we would promote further studies, subject to a randomised controlled clinical trial methodology to compare cost-effective psychological intervention delivery methods.

## Competing interests

The authors declare no competing interests.

## Compliance with ethical standards

Informed consent was obtained from all individual participants included in the study.

## Funding

No funding.

## Figures and Tables

**Table 1.** Patients’ characteristics.

*N*	51
Age (years)	Mean (sd)
	54.1 (13.4)
	*N* (%)
<50	16 (31.4)
50–59	17 (33.3)
≥60	18 (35.3)
Diagnosis	*N* (%)
Cancer ovary	24 (48.0)
Cancer uterus	15 (30.0)
Cancer vulva	5 (10.0)
Other diagnosis*	6 (12.0)
Stage of disease	*N* (%)
First diagnosis	37 (72.6)
Recurrence	14 (27.4)
Number of psy sessions	*N* (%)
1	26 (51.0)
2	17 (33.2)
3	3 (5.9)
4	1 (2.0)
5	1 (2.0)
6	1 (2.0)
8	2 (3.9)
Type of intervention^	*N* (%)
Assessment	32 (64.0)
Empowerment	18 (36.0)
Other reason from cancer (6 months follow-up)	*N* (%)
No	35 (68.6)
Yes	16 (31.4)

* benign disease.

**Table 2.** Variation of distress: comparison between subgroups.

	Distress pre-surgery				Distress post-surgery (6-month follow-up)			
	*N*	Mean (SD)	Test value	*p*-value	*N*	Mean (SD)	Test value	*p*-value
ALL	51	7.69 (2.0)			44	6.95 (2.6)		
Age (years)				0.07				0.80
< 50	16	8.1 (1.9)	5.221^		12	7.0 (2.7)	0.429^	
50;59	18	8.2 (1.6)			17	7.2 (2.2)		
≥ 60	17	6.7 (2.3)			15	6.5 (2.9)		
Diagnosis				0.81				0.95
Cancer ovary	24	7.6 (2.1)	0.942^		20	7.0 (2.1)	0.327^	
Cancer uterus	15	7.3 (2.4)			13	7.0 (2.4)		
* Cancer vulva*	5	8.2 (1.6)			5	8.2 (1.6)		
Other diagnosis*	6	8.5 (1.0)			5	8.5 (1.0)		
Stage of disease				0.02*				0.46
First diagnosis	37	8.0 (1.9)	259.5“		31	6.74	669.5”	
Recurrence	14	6.6 (2.1)			13	7.46		
Number of interviews				0.81				0.45
1	26	7.5 (2.0)	0.95^		22	6.5 (2.8)	2.62^	
2	17	7.7 (2.3)			15	7.2 (2.6)		
3	3	8.6 (1.1)			2	9.0 (1.4)		
≥4	5	7.8 (1.9)			5	6.8 (1.4)		
Type of intervention				0.42				0.09
Assessment	32	7.8 (2.0)	420.0”		26	7.5 (2.3)	336.0”	
Empowerment	18	7.3 (2.1)			18	6.1 (2.8)		
Other reason from Cancer (6 months follow-up)				0.87				0.007**
No	35	7.7 (1.9)	408.5”		28	6.1 (2.7)	521.0”	
Yes	16	7.5 (2.3)			16	8.3 (1.7)		

^ Kruskal–Wallis test

“ Wilcoxon’s test

* *p* < 0.05

** *p* < <0.01

**Table 3.** Satisfaction and perceived utility of the consultation: comparison between subgroups at 6-month follow-up.

	Hospitalisation				Personal issues				Returning home			
	*N* Yes	*X*^2^ value	Mean utility	Test value (Kw or W)	*N* Yes	*X*^2^ value	Mean utility	Test value (Kw or W)	*N* Yes	*X*^2^ value	Mean utility	Test value (Kw or W)
Age (years)		3.07		2.36^		0.43		0.87^		4.63		3.63^
< 50	7		9.4 (1.1)		7		8.8 (1.2)		6		8.6 (1.5)	
50–59	14		8.5 (1.2)		8		9.1 (1.1)		14		8.0 (1.1)	
≥ 60	10		9.0 (1.2)		8		8.5 (1.4)		10		8.8 (1.8)	
Diagnosis		2.73		4.74^		3.28		3.40^		6.03		0.34^
Cancer ovary	12		8.5 (1.2)		9		9.1 (1.1)		14		8.0 (1.7)	
Cancer uterus	9		9.0 (1.3)		8		8.5 (1.4)		9		8.3 (1.3)	
Cancer vulva	4		10.0 (0.0)		2		10 (0.0)		4		9.2 (0.9)	
Other diagnosis*	5		8.8 (1.3)		3		8.6 (0.5)		2		8.5 (0.7)	
Stage of disease		3.63		106.5“		8.90**		40.5”		2.54		345.5”
First diagnosis	23		9.0 (1.1)		19		8.9 (1.2)		23		8.3 (1.5)	
Recurrence	8		8.5 (1.4)		4		8.5 (1.2)		7		8.7 (1.2)	
Number of interviews		4.14		2.41^		3.23		0.18^		1.85		4.09^
1	13		8.6 (1.2)		10		8.7 (1.4)		14		7.8 (1.6)	
2	9		9.0 (1.3)		7		9.0 (1.1)		9		8.5 (1.1)	
3	2		10.0 (0.0)		1		9.0 (0.0)		2		8.5 (2.1)	
≥ 4	5		8.8 (1.3)		3		8.6 (1.5)		4		9.5 (1.0)	
Type of intervention		5.37		287.5“		1.93		95.5”		0.61		188.0”
Assessment	18		8.8 (1.2)		14		9.0 (1.2)		17		8.5 (1.3)	
Empowerment	13		8.9 (1.1)		9		8.5 (1.2)		13		8.1 (1.7)	
Other reason from Cancer (6 months follow-up)		0.29		159.5”		7.13*		90.5”		4.76		325.5”
No	21		8.9 (1.7)		15		8.9 (1.1)		22		8.3 (1.2)	
Yes	10		8.9 (1.3)		8		8.6 (1.5)		8		8.5 (2.1)	

^ Kruskal–Wallis test

“ Wilcoxon’s test

* *p* < 0.05

** *p* < 0.01
